# Impact of Acute Versus Chronic Unilateral Hearing Loss on Head Movement in a Novel Binaural Task

**DOI:** 10.1002/lary.70034

**Published:** 2025-08-11

**Authors:** Madison V. Epperson, Obada Abdulrazzak, Gerilyn Jones, Nadine I. Ibrahim, Renee M. Banakis Hartl

**Affiliations:** ^1^ Department of Otolaryngology‐Head & Neck Surgery University of Michigan Ann Arbor Michigan USA; ^2^ University of Arizona College of Medicine Phoenix Arizona USA

**Keywords:** acute versus chronic hearing loss, hearing loss duration, localization, single‐sided hearing loss, speech‐in‐noise performance, unilateral hearing loss

## Abstract

**Objectives:**

Individuals with single‐sided deafness (SSD) may develop adaptive listening strategies with head movement patterns to optimize monaural localization and speech‐in‐noise understanding. Granular understanding of adaptive behaviors may better inform rehabilitation for SSD. We aimed to characterize head movements during a combined localization and speech‐in‐noise task to understand how adaptive behaviors emerge.

**Methods:**

Prospective study from a tertiary referral center with 16 subjects with normal hearing (NH) and 15 SSD subjects. Sentences were played in a semi‐anechoic chamber from one of 24 speakers in a 360° azimuthal configuration with a variable signal‐to‐noise ratio. Head position was captured via an electromagnetic tracking system. NH subjects completed the task twice, once with a deeply‐seated earplug and supra‐aural earmuff to simulate acute unilateral hearing loss and once unoccluded. Outcome measures included localization accuracy (mean absolute error, slope across targets), head movement (onset delay, total response time, total head displacement), and speech‐in‐noise performance.

**Results:**

Unoccluded NH subjects displayed accurate localization, minimal movement delay, rapid response time, low total head displacement, and high speech‐in‐noise percent correct compared to the occluded condition and SSD subjects. Localization accuracy and SIN performance were comparable between NH occluded and SSD; however, the groups had distinct head movement patterns.

**Conclusions:**

Acute unilateral hearing loss leads to sharp declines in localization accuracy and speech‐in‐noise performance. In SSD, difficult listening conditions may prompt the development of distinct head movement patterns over time. This work provides key initial insight into adaptive listening strategies that individuals with SSD may acquire and utilize in complex listening environments.

**Level of Evidence:**

3

## Graphical abstract

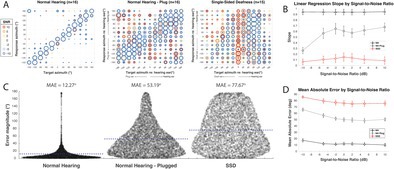

## 1 Introduction

Moderate or worse unilateral hearing loss affects over 3.5 million Americans, comprising 1.5% of individuals [1, 2]. The most severe form of unilateral hearing loss, single‐sided deafness (SSD) is associated with higher rates of stress and anxiety, with decreased quality of life [3]. They face immense challenges in complex listening environments that rely on the benefits provided by binaural hearing [4, 5, 6, 7]. Due to lack of acoustical binaural cues, they struggle with two specific tasks: sound localization and understanding speech‐in‐noise (SIN) [8, 9, 10]. Prior research has shown that individuals with SSD use a combination of spectral pinna cues in combination with head movements to capitalize on the head shadow effect to attempt to localize [11].

Prior work has confirmed that in SSD there is significant inter‐individual variability in sound source localization [11, 12, 13], possibly related to the structural plasticity of the auditory cortex [13]. Some individuals perform better than expected, particularly at high frequencies where shorter wavelengths might afford access to complex monaural cues generated by head‐related transfer functions and specific head movement patterns [12]. Although head movement is often restricted for testing purposes, this is not reflective of the everyday listening experience, where individuals are permitted to move their heads freely. There is evidence that head movement is important in this population and improves SIN and localization [14, 15, 16, 17, 18]. These findings suggest that those with SSD may develop adaptive listening strategies with specific head movement patterns to optimize monaural localization abilities and SIN understanding. Granular understanding of these adaptive behaviors in complex listening environments may better inform aural rehabilitation in SSD. With this in mind, the objectives of this study were to characterize localization ability, speech understanding in noise, and adaptive head movement listening strategies during a novel, combined localization and SIN task for those with severe unilateral hearing loss. We compared acute single‐sided hearing loss (with ear plugging) to established SSD to better understand how adaptive behaviors might emerge.

## 2 Materials and Methods

### 2.1 Participants

Subjects were included if they were between 18 and 75 years of age and demonstrated either hearing sensitivity within normal limits in both ears (normal hearing) or a severe to profound unilateral sensorineural hearing loss (SNHL) with a four‐frequency PTA ≥ 80 dB HL and normal or near‐normal hearing in the contralateral ear (SSD). In our testing paradigm, normal hearing thresholds were defined as 25 dB HL or better across octave frequencies from 250 to 8000 Hz. Normal hearing (NH) participants had a hearing screening in the lab prior to testing if an audiogram performed within the last 12 months was not available. Exclusion criteria included lack of English language fluency, presence of neurodegenerative disease, or presence of known retrocochlear pathology in the normal hearing ear of SSD participants or in either ear of the NH participants. NH participants were recruited via the University of Michigan Health Research Recruitment database (https://umhealthresearch.org). SSD participants were recruited in Otology clinic or via phone if a known SSD patient from clinic. This study was approved by the University Institutional Review Board HUM00190678.

### 2.2 Combined Localization and Speech‐In‐Noise Testing Paradigm

**FIGURE 1:**
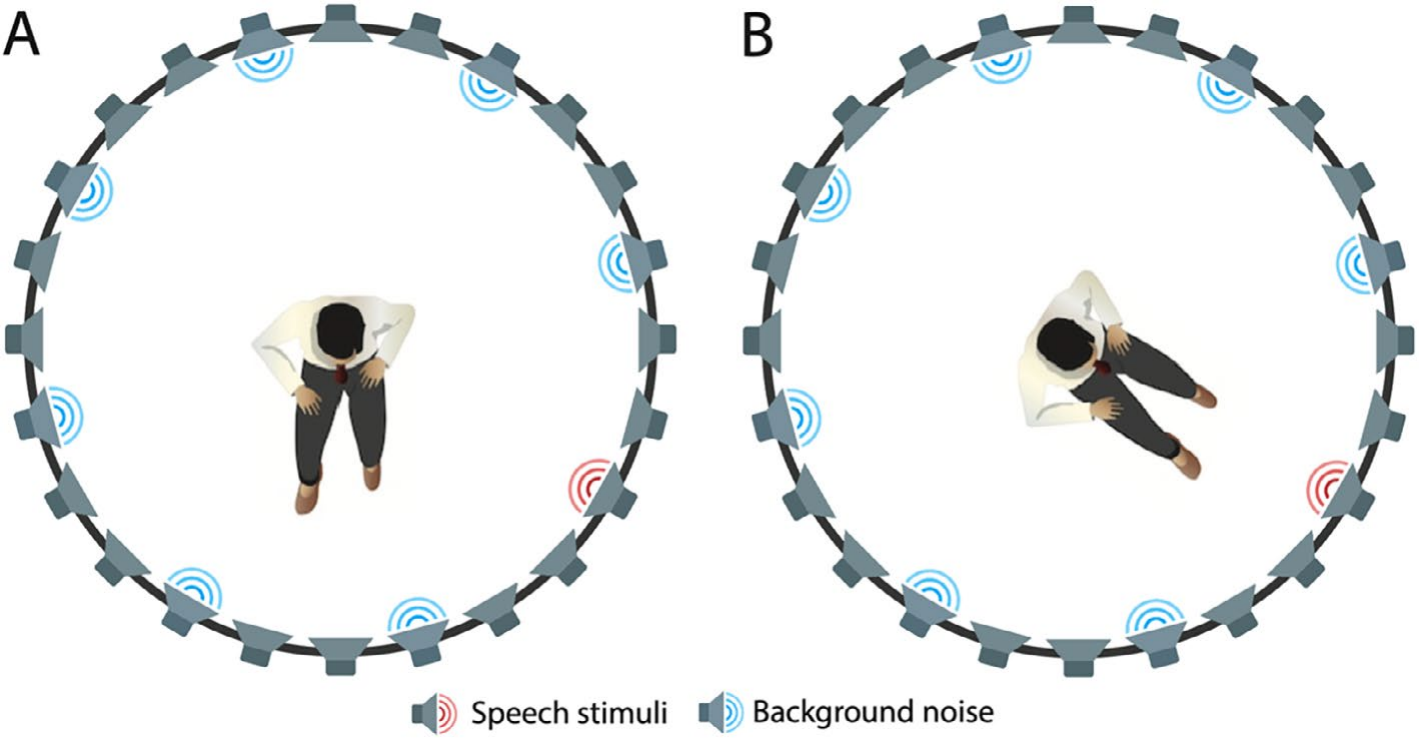
Experimental setup indicating one of twelve speaker configurations. (A) Participant begins zero degrees azimuth. (B) They move naturally to optimize listening, then turn to where they perceive the stimulus origin. [Color figure can be viewed in the online issue, which is available at www.laryngoscope.com]

Testing was completed in a darkened semi‐anechoic chamber with 24‐speakers spaced equally in a 360° azimuthal configuration at the level of the pinnae. As described previously [19, 20], a non‐target orienting speech stimulus was delivered, with both low and high frequency components, “Hey, look over here. I'm over here. Look over here.” This was immediately followed by Harvard IEEE sentences presented at 70 dB SPL from one of twelve randomly selected speakers in the presence of diffuse pink noise with variable signal‐to‐noise ratios (SNR) of −10, −5, −2, 0, 2, 5, and 10 dB (Figure 1). Participants began the task with facing 0° azimuth. They were allowed to move naturally to optimize listening. After listening, they turned towards the perceived stimulus location and indicated their position with a button press while also repeating the target sentence. LED lights were present in the position of all speakers and lit up after the participant indicated stimulus location to control for the possibility of differential eye, head, and body movement. Patients returned to 0° azimuth after each trial, guided by an LED light. Head movements were recorded via a Fastrak electromagnetic tracking system (Polhemus, Colchester, VT). Custom‐built stimulus presentation and response acquisition software written in MATLAB (R2019b; The Mathworks Inc., Natick, MA, United States) were used to generate the stimuli, drive the speakers, and record head position from the electromagnetic tracking system. Video monitoring was utilized to ensure protocol compliance.

### 2.3 Testing Conditions

NH participants underwent testing with a simulated unilateral acute maximal conductive hearing loss (NH‐plug) via a combination of a deeply seated foam earplug (3M E‐A‐R soft yellow neon plugs, 33 dB noise reduction rating) and a supra‐aural earmuff (3M PELT OR X3A Ear Muffs, 28 dB noise reduction rating). The laterality of plugging was determined randomly with an online randomization tool. They also completed unoccluded testing (NH‐unoccluded). The testing order of plugging condition was also randomized. To mitigate the learning effect, a minimum of 7 days between testing conditions was enforced. SSD subjects completed testing without personal amplification devices or implants. Each speaker‐SNR combination was repeated 5 times, totaling 420 trials per condition, which took on average 2.5–3.5 h to complete. Participants were given multiple opportunities for breaks and rests between trials.

### 2.4 Outcome Measures and Analysis

MATLAB was used for statistical analysis. Comparisons were assessed at the *α* = 0.05 level. Post hoc Bonferroni corrections were utilized in analyses with multiple comparisons. Repeated‐measures (rm) analysis of variance (ANOVA) was used to compare the within‐group impact of plugging on control subjects and analyze the effects of SNR and speaker target locations/target azimuth (TargAz) on measures of interest for control, allowing for controlling individual differences and reducing the error variance; repeated measures provide a more precise estimate of the effects by considering within‐subject correlations. A three‐way ANOVA was used to analyze the effects of hearing status (HS), SNR, and TargAz on measures of interest to compare performance between NH‐plug and SSD groups. Post hoc Tukey honest significance difference (hsd) was used when applicable to assess for significance of factor interactions.

#### 2.4.1 Analysis

##### 2.4.1.1 Localization Accuracy

Localization accuracy was assessed with two outcome measures: mean absolute error (MAE) and the slope of the line of best‐fit across targets on a localization accuracy plot. Use of slope as a metric of localization accuracy in addition to MAE allows characterization of performance more broadly across the entire azimuthal array [21]. Slope was calculated for each subject using robust linear regression. When considering slope as a measure of localization accuracy, a slope closer to one indicates greater accuracy.

#### 2.4.2 Head Movement Dynamics

Head movement dynamics were characterized with three outcome measures: movement onset delay, total response time, and total head displacement. Onset delay was the time elapsed from the start of the trial until a threshold movement of at least 15°. Total response time was the duration from the movement onset until the participant registered perceived stimulus location with a button click. Total head displacement was the total magnitude of head movement in degrees.

#### 2.4.3 Speech‐In‐Noise Performance

SIN performance was evaluated as percent correct of target stimuli. The effect of hearing status, plugging, TargAz location, and SNR on performance scores was evaluated.

## 3 Results

This study included 31 subjects, 15 with SSD and 16 NH who underwent simulation of acute unilateral hearing loss. Participant characteristics are displayed in Table 1.

**TABLE 1:** Participant characteristics.

	Single‐sided deaf (*n* = 15)	Normal hearing controls (*n* = 16)
Female, *n* (%)	10 (66.7)	8 (50.0)
Age^a^	61.3 ± 8.1	40.4 ± 17.3
Duration of deafness, years^a^, ^b^	2.2 ± 2.3	Not applicable
Etiology of deafness, *n* (%)	1 (6.7)	Not applicable
Sudden sensorineural	7 (46.7)	
Labyrinthitis	3 (20.0)	
Congenital	2 (13.3)	
Following SSCD^c^ repair	1 (6.7)	
Tumor^d^	1 (6.7)	
Trauma	1 (6.7)	

^a^ Mean ± standard deviation.

^b^ Minimum = 0 years; maximum = 8 years.

^c^ SSCD‐ superior semicircular canal dehiscence.

^d^ Following translabyrinthine approach for endolymphatic sac tumor.

### 3.1 Localization Accuracy

#### 3.1.1 Slope (m)

**FIGURE 2:**
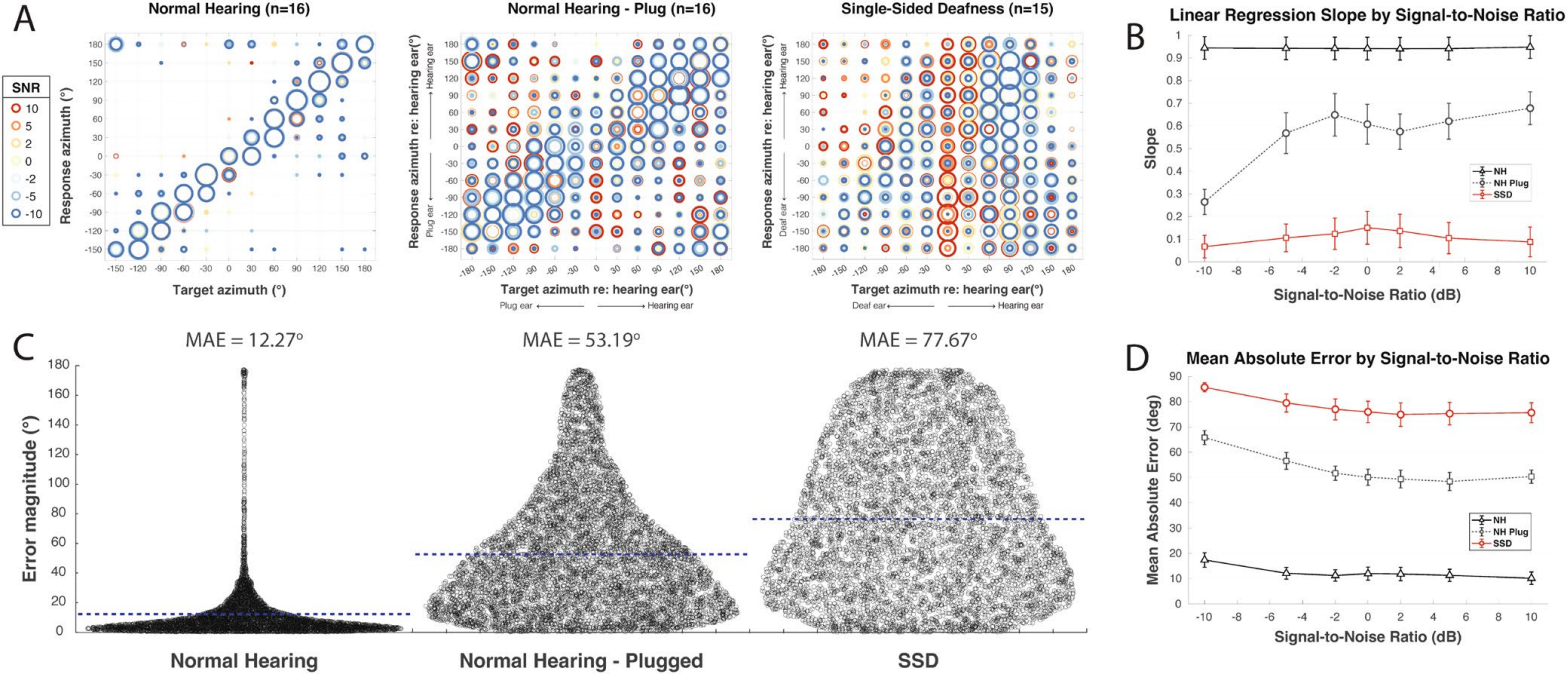
Localization accuracy. (A) Bubble plots displaying target azimuth and response location, corrected for the side of deafness (positive values towards the normal hearing ear). The cooler colors represent lower signal‐to‐noise ratios (SNR), more challenging listening conditions. (B) Plot demonstrating how slope changes with variable SNRs for each group. (C) Error plots depicting the mean absolute error (blue line) with the width of the plot demonstrating the number of responses at that error magnitude. (D) Plot demonstrating how mean absolute error changes with variable SNRs. [Color figure can be viewed in the online issue, which is available at www.laryngoscope.com]

Localization accuracy by slope is shown in Figure 2A; NH‐unoccluded subjects localize well across SNR with high average slope (*m =* 0.91). Accuracy significantly deteriorates in the NH‐plug condition (*p* << 0.0001), indicated by reduced slope (*m =* 0.52). Localization is also significantly poor for the SSD cohort compared to the NH‐plug group with *m =* 0.07 (*p* << 0.0001).

The effect of SNR on performance slope is shown in Figure 2B, which was statistically significant for both group comparisons (*p* < 0.0417). In the NH‐unoccluded group, slope was close to one, even in the more challenging listening conditions. The NH‐plug condition exhibited very low slopes in the most challenging conditions (−10 SNR) improving to *m =* 0.65 at a maximum. The SSD group had the worst performance, with a maximum average slope of *m =* 0.15. There was not a significant effect of the interaction of hearing status and SNR intergroup comparison (two‐way ANOVA).

#### 3.1.2 Mean Absolute Error (MAE)

MAE for cohorts is shown in Figure 2C. The NH‐unoccluded group demonstrated low MAE (12.27°). MAE is higher with NH‐plug (53.19°) and even higher for SSD (77.67°). Repeated measures ANOVA examining the effect of plug status on MAE demonstrated a very strong effect of plug status (*p* << 0.0001) and significant independent effects of both SNR and TargAz (*p* << 0.0001), as well as a significant three‐way interaction between SNR, TargAz, and plug status (*p* << 0.0001). When comparing NH‐plug to SSD, three‐way ANOVA revealed NH‐plug group status, higher SNR (easier listening condition), and target location closer to center were associated with lower MAE (*p* << 0.0001). There was a significant interaction of HS and TargAz (*p* << 0.0001), but no other two‐ or three‐way significant interactions (*p* > 0.5005).

The specific effect of SNR on MAE is depicted in Figure 2D. In the NH group, MAE was low, even in the most challenging listening conditions. In the other two groups, MAE is higher in the more challenging conditions and decreases slightly, but remains elevated in the easier listening conditions.

### 3.2 Head Movement Analysis

#### 3.2.1 Onset Delay

**FIGURE 3:**
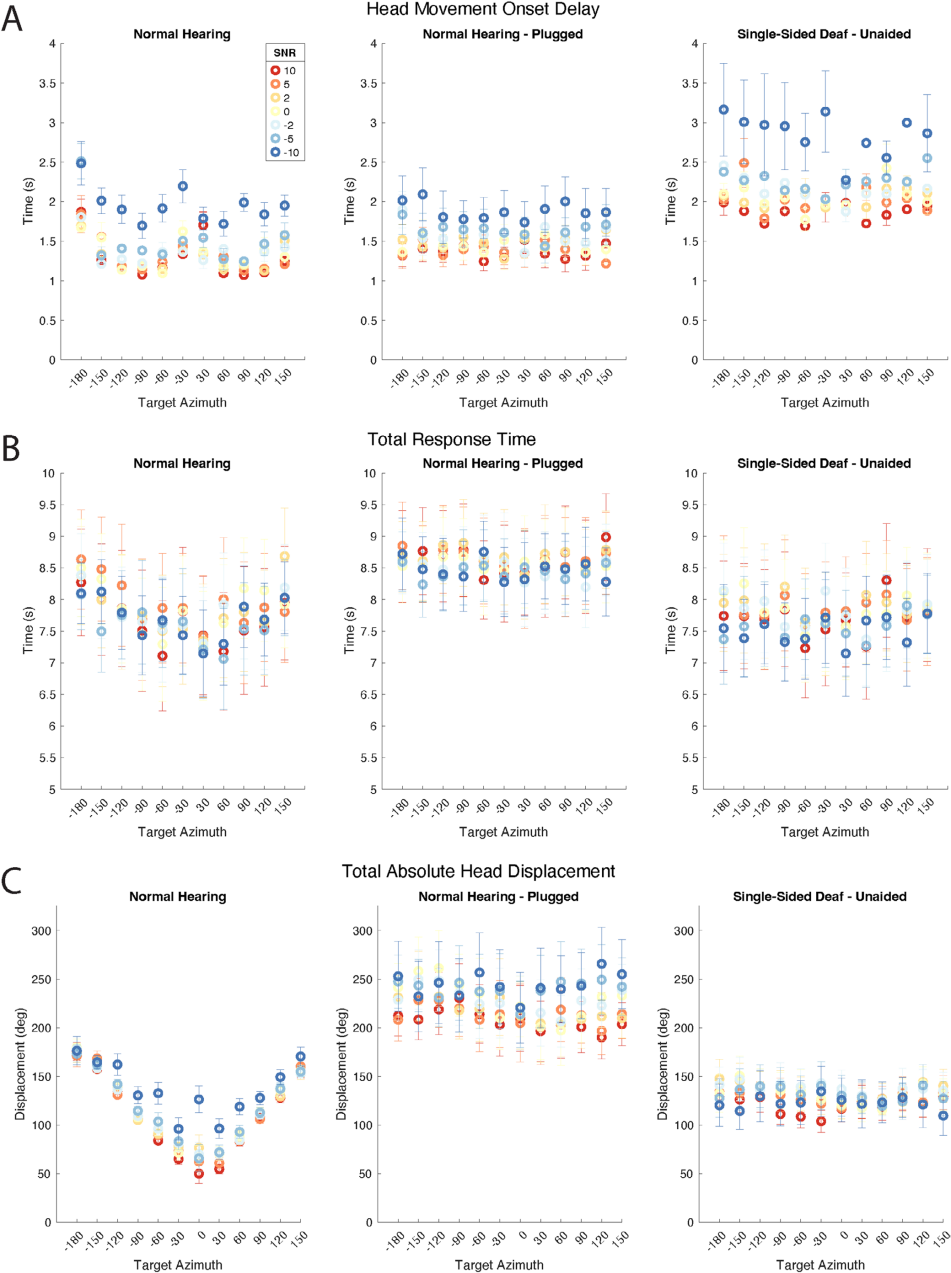
Head movement analysis by target azimuth. (A) Head movement onset delay with variable signal‐to‐noise ratios (SNR). (B) Total response time by SNR. (C) Total absolute head displacement by SNR. [Color figure can be viewed in the online issue, which is available at www.laryngoscope.com]

**FIGURE 4:**
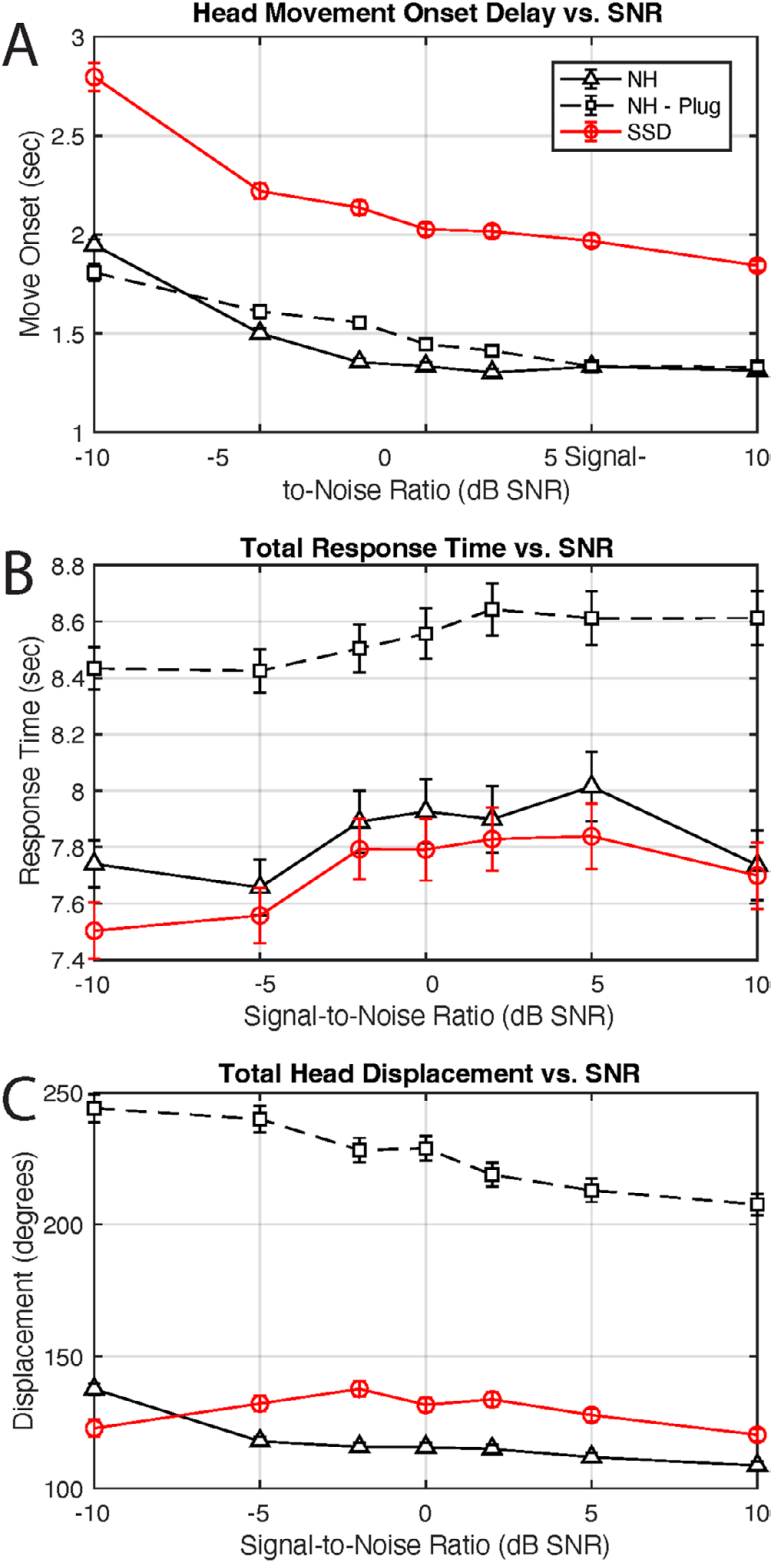
Head movement analysis by signal‐to‐noise ratio (SNR). (A) Head movement onset delay. (B) Total response time. (C) Total absolute head displacement. [Color figure can be viewed in the online issue, which is available at www.laryngoscope.com]

Head movement onset delay is displayed by TargAz (Figure 3A) and by SNR (Figure 4A) for each group. SSD subjects displayed greater onset delays compared to NH subjects, in both occluded and unoccluded conditions. As SNR became more favorable, movement onset delays decreased in all groups. An rmANOVA revealed a significant overall effect of plug status (*p* = 0.0045) and a significant main effect of TargAz (*p* << 0.0001), with no effect of SNR or the interaction (*p* > 0.22954). A three‐way ANOVA demonstrated that NH status, higher SNR, and the HS:SNR interaction were associated with reduced onset delay (all *p* < 0.0195). TargAz as a main effect and the remaining interactions were non‐significant (*p* > 0.4544).

#### 3.2.2 Total Response Time

Total response time for each group is displayed by TargAz (Figure 3B) and by SNR (Figure 4B). Unlike the other measures, total response time is relatively stable across SNR. Neither SNR (*p* > 0.7826) nor TargAz (*p* > 0.2711) significantly affected response time independently by either rmANOVA evaluating the impact of plugging or ANOVA comparing NH‐plug to SSD. Interactions were also not significant (*p* > 0.8401). Notably, the NH‐plug condition demonstrates the highest average response time of any group, greater than NH‐unoccluded (*p* = 0.0006) and SSD (*p* << 0.0001).

#### 3.2.3 Total Absolute Head Displacement

Total absolute head displacement in degrees is depicted by TargAz (Figure 3C) and by SNR (Figure 4C) for all groups. NH individuals exhibit precise and efficient head displacement, exhibited by the v‐shaped configuration that results from expected increasing head displacement as the speaker location is further from the starting position (see Figure 3C). With lower SNR (more challenging listening condition), indicated by the dark blue circles, head displacement is slightly less precise. Plugged subjects have significantly elevated displacement across SNR compared to the unoccluded condition (*p* << 0.0001). SSD subjects demonstrate relatively uniform displacement, irrespective of target location, significantly less than with the acute hearing loss in plugged subjects (*p* << 0.0001). An rmANOVA assessing the impact of plugging in the NH group and three‐way ANOVA comparing NH‐plug to SSD revealed SNR was a marginally significant factor only between the latter groups (*p* = 0.0467), and TargAz, SNR (for NH group) and interactions were not significant factors influencing total displacement (*p* > 0.1527).

The most striking response pattern is the substantially elevated displacement associated with plugging, just as the plug condition resulted in the longest average response time of any group; in the setting of an acute unilateral hearing loss, individuals take longer and move their heads more in their attempts to localize and discriminate speech.

### 3.3 Speech‐In‐Noise

**FIGURE 5:**
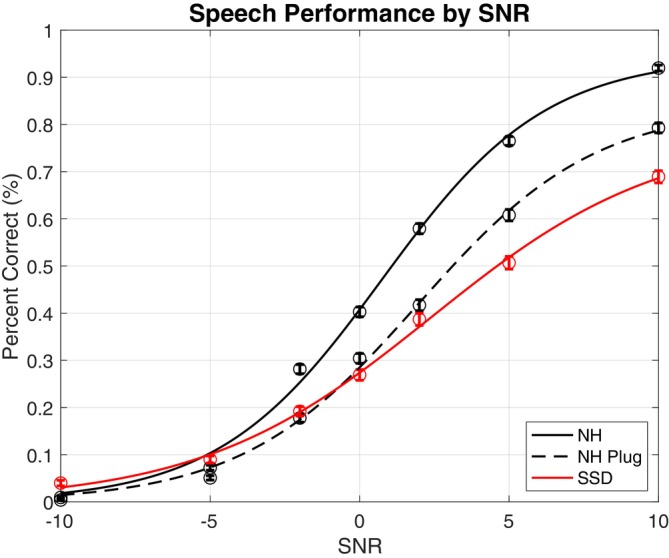
Speech‐in‐noise percent correct across signal‐to‐noise ratios. The dots on the graphs represent group mean performance at each SNR. Error bars indicate the standard error of the mean. [Color figure can be viewed in the online issue, which is available at www.laryngoscope.com]

As illustrated in Figure 5, in more challenging SNR conditions, all groups exhibit nearly equivalent poor performance. As SNR increases, performance accuracy improves for all groups. However, performance in the NH‐plug (*p* << 0.0001) and SSD (*p* < 0.0001) groups remains inferior to the NH‐unoccluded groups at all SNRs greater than −5 dB SNR. Three‐way ANOVA revealed a significant main effect of higher SNR (*p* << 0.0001) and target location close to the center (*p* = 0.0038) associated with better SIN performance, as well as significant two‐ and three‐way interactions (*p* << 0.0001). Only SNR demonstrated a main effect on speech performance when evaluating the effect of plugging (*p* << 0.0001), while Targ Az and the interaction were non‐significant (*p* > 0.1380).

## 4 Discussion

Individuals with SSD face challenges in difficult listening environments due to a lack of binaural cues that allow for precise localization of sound and improved speech understanding in noise [15, 16, 18]. In place of binaural cues, it is theorized that individuals with SSD use a combination of complex monaural level cues and pinna and head movement‐related spectral shape cues [22], but the utilization of these cues is ambiguous and poorly understood. This is in part due to a lack of testing environments that mimic the complex multi‐talker, multi‐location listening scenarios encountered by these individuals in the real world. Furthermore, in most testing protocols, head movements are restricted, limiting understanding of how individuals may learn to move their heads in these complex environments to optimize their hearing performance.

To address these questions, we sought to investigate adaptive head movement patterns during a novel, combined localization and SIN task in individuals with severe unilateral hearing loss where head movement was unrestricted and carefully tracked. By comparing patients with a simulated acute maximal loss to SSD, we aimed to elucidate how head movement patterns might change over time. Better understanding of how head movement patterns develop in SSD to optimize localization and SIN understanding may inform cochlear implant device programming strategies [6, 8, 10, 14, 23]. Acute unilateral hearing loss leads to sharp declines in localization accuracy and SIN performance with changes in head movement patterns that are distinct from those with established SSD.

### 4.1 Localization

Prior research has demonstrated that localization in SSD and in individuals with simulated unilateral hearing loss is poor [17, 22, 24, 25]. In our study where head movement was unrestricted and noise was diffuse, localization data largely align with prior studies. We found that localization ability significantly deteriorates in the NH‐plug condition, deteriorating even further in those with chronic SSD.

It is interesting to consider that the NH‐plug cohort localized more accurately by both metrics when compared with the established SSD group. These findings are similar to those observed by Kim et al. [26], but in contrast to those observed by Pastore et al. [17]. One potential explanation for the discrepancy is the type of hearing impairment; the NH‐plug group represents an acute maximal conductive loss compared to the severe to profound sensorineural loss seen in the SSD group. The slightly improved performance in the NH‐plug cohort, although still poor, may suggest that they are still accessing some acoustic information that SSD subjects cannot, particularly significantly diminished high‐frequency level difference cues. With relatively high presentation levels (70 dB SPL), it is possible that there may be some stimulation of the plugged ear via bone conduction stimulation.

An alternative possibility may be that over time, individuals with SSD gain experience with the inability to localize, resulting in less engagement and less effort to use potentially limited available cues. Although our cohort did not receive visual feedback or task‐specific training known to improve localization abilities due to gradual reweighting of cues [22, 25], a component of learning by the NH‐plug or SSD individuals cannot be excluded.

### 4.2 Head Movements

It has been demonstrated in prior study that allowing NH individuals to move their heads during localization tasks improves accuracy [18, 27]. Head movement reduces errors in the front‐back and left–right dimensions for NH‐plug and SSD [17]. In SIN tasks, individuals with asymmetric hearing loss will move their heads to maximize the level of the target signal [15]. Our study was unique in that we aimed to characterize multiple objective metrics of head movement in a complex listening task.

In our granular analysis of head movement dynamics, we found that NH subjects move their head efficiently with minimal movement onset delay, modest total response time, and head displacement proportionate to target azimuth. An acute unilateral hearing loss (NH‐plug) leads to significantly elevated absolute head displacement and total response time, greater than that of the chronic SSD cohort; with an acute unilateral hearing loss, individuals take longer and move their head more in their attempts to localize and discriminate speech. SSD subjects had distinct movement patterns from the NH‐plug group, with greater movement onset delay but reduced total response time and absolute head displacement; SSD subjects waited longer to begin moving but moved less and more quickly than NH‐plug. This raises the question of whether a greater onset delay is a learned behavior in SSD.

### 4.3 Speech‐In‐Noise

Our study assessed speech understanding from multiple different targets in the setting of diffuse background noise, permitting head movements, to simulate a real‐world listening scenario. Testing in a complex environment was critical as prior data suggests that the benefit of binaural hearing for SIN is especially relevant when there are multiple interferers at different locations from the target [28]. We found that in the more difficult SNR conditions, all subjects, regardless of hearing status, exhibit nearly equivalently poor performance. As the SNR increases, performance accuracy improves for all groups, but along different trajectories, with all groups achieving different maximums: NH performance worsens with plugging, and SSD subjects perform more poorly than NH plugged subjects. These findings are consistent with prior literature demonstrating that a stimulated unilateral acute maximal conductive hearing loss significantly decreases SIN performance [10, 24]. Our research furthers prior literature in demonstrating the consistency of these findings even in a complex combined SIN and localization task.

### 4.4 Limitations and Future Directions

Our results should be interpreted with consideration of the study limitations. Given the challenges of recruiting and efficiently testing patients with an acute sensorineural loss, the simulated acute maximal unilateral hearing loss in our population was conductive in nature with a plug and supra‐aural earmuff. We recognize the distinct entities of conductive versus sensorineural loss and the possibility that those with a maximal conductive loss may still have access to certain diminished but present acoustic cues unavailable to those with chronic SSD due to sensorineural loss. However, our study still reveals unique head movement patterns in the acute loss group, shedding light on how head movement patterns *may* change over time when compared to those with established SSD. It further provides elucidation of head movement patterns in SSD.

Separately, it must be considered if isolated tasks of binaural hearing without contextual clues, such as in most laboratory environments, accurately represent real world behaviors where other multimodal cues, namely visual cues, are readily accessible. It may be possible that performance differs in real world environments; this may explain discrepancies in objectively poor performance on lab testing and subjective benefits seen in patient questionnaires. Lastly, although significant differences are seen in binaural metrics amongst tested groups in our study, it is unknown what truly meets the threshold for clinically significant differences.

## 5 Conclusions

Head movement dynamics change over time with sudden unilateral hearing loss. In acute maximal conductive hearing loss, individuals take longer and move their head more. With established SSD, the pattern is much different, with increased movement onset delay but reduced absolute head displacement and total response time. Hearing capability fundamentally affects how other factors (including SNR and target location) influence performance. These results allow for a better understanding of head movement dynamics in difficult listening conditions in SSD, laying the foundation for future efforts to improve device programming and aural rehabilitation strategies aimed to optimize listening in this population.

## Conflicts of Interest

The authors declare no conflicts of interest.
